# Theoretical Study on the Dynamic Behavior of a Plate-Like Micro-Cantilever with Multiple Particles Attached

**DOI:** 10.1371/journal.pone.0151821

**Published:** 2016-03-29

**Authors:** Liang Zhao, Fei Wang, YanLing Zhang, Xuezeng Zhao

**Affiliations:** School of Mechanical and Electronic Engineering, Harbin Institute of Technology, Harbin 150001, China; Massachusetts Institute Of Technology, UNITED STATES

## Abstract

In this study, the dynamic characteristics of a plate-like micro-cantilever beam attached with multiple concentrated masses are studied. The vibration modes of the cantilever plate are represented by combinations of beam functions. Using classical mechanics (the effect of size is not considered) and the corrected Cosserat’s theorem (the effect of size is considered), we employ the Lagrange equations to establish a dynamic model of the plate-like micro-cantilever beam attached with multiple concentrated masses. The accuracy of the model proposed in this paper is verified by comparing with the results of published literature. Then, the natural frequencies of the cantilever plates are calculated with self-compiled algorithms, and the results of the plates with 1–5 masses are displayed. The results are in high accordance with the exact solution, and all errors are within 0.5%. The analysis shows that the proposed model and analysis method converges quickly and is highly efficient. In addition, the effects of characteristic lengths, Poisson's ratios and plate thickness on the micro-cantilever plate’s resonant frequency for the first five modes are analyzed.

## Introduction

Micro-mass detection using a micro-cantilever beam sensor has been widely applied to measure one single cell or molecule with high accuracy[1,2,3] since it was first proposed in 1995 [4,5]. Typically, the measurement parameters of the dynamic response of the micro-cantilever beam sensor are used to establish the dynamic model of the micro-cantilever beam with multiple ultra-micro-masses attached[6,7], and from this inverse solution, the mass parameters of the attached particles can be obtained [8].

Current research on micro-cantilever sensors is concentrated on single-beam with multiple attached particles [9,10,11] and, alternatively, single-beam with uniformly distributed molecules attached [12]. However, for the detection of individual adsorbed masses of a plate-like micro-cantilever beam, two limitations need to be noticed 1) Most micro-cantilever sensors in a practical application have a plate-like structure; however, in most cases their dynamic behaviors are analyzed using beam theory, and dynamic analysis with plate theory has rarely been reported. 2) The thickness of the practical micro-cantilever sensor is on the micrometer scale, and as a result, the effect of size cannot be neglected, but studies on this effect are quite scarce.

In this study, plate theory is utilized to establish the dynamic model of the plate-like micro-cantilever sensor with multiple concentrated masses attached considering the size effect. The computed results are then compared with related references to verify the efficacy of the improvements of the established model in our study. Additionally, the natural frequencies of a widely applied type of rectangular micro-cantilever sensor with multiple concentrated masses attached are calculated using a self-compiled algorithm, and the results for sensors with 1–5 masses are presented.

## Materials and Methods

**Fig 1:**
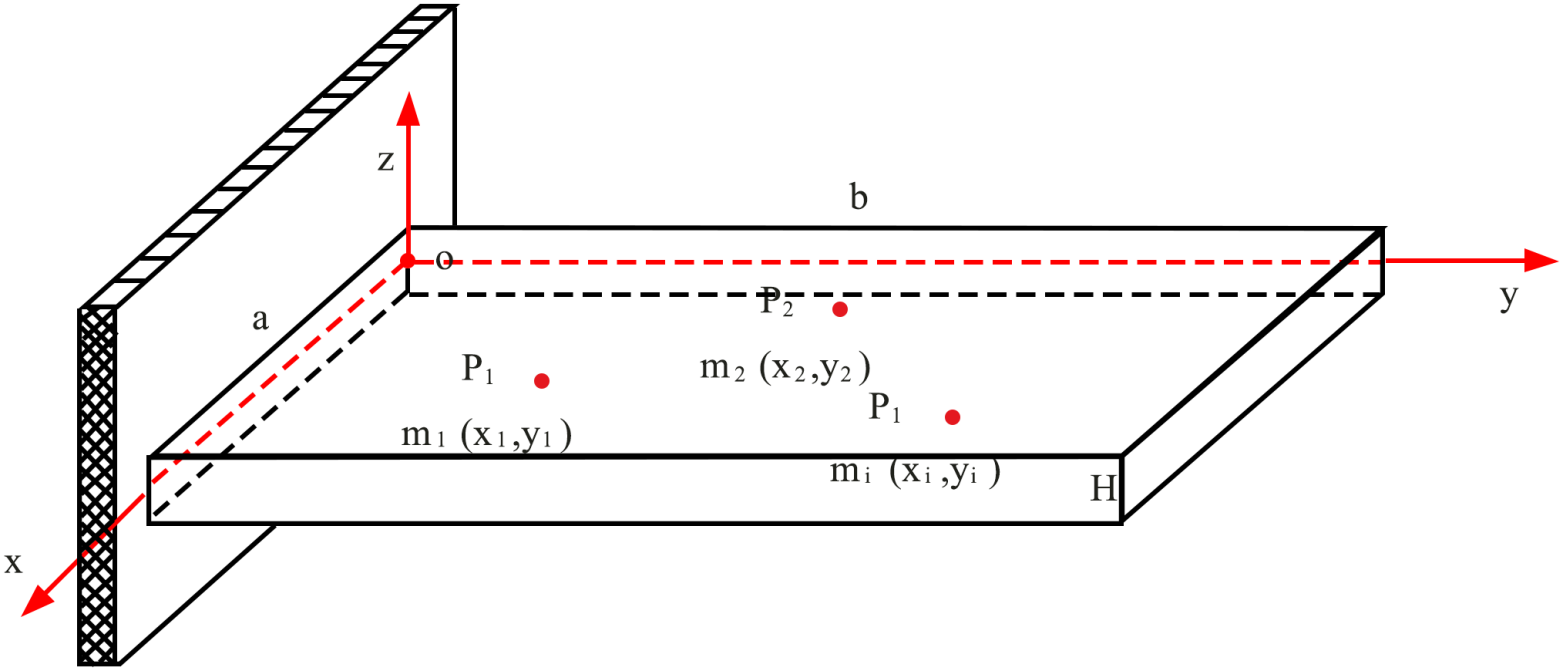
Model of the plate-like micro-cantilever beam with concentrated masses attached, which are dispersed in different locations.

A micro-cantilever plate with N concentrated masses attached is set as the research object in this study. The masses are spherical particles of different substances with known material properties, diameters (mass is known) and locations on the beam (the distributions of the particles along the length and width of the beam are known). The size and material properties of the plate are also known (the scale of the length of the plate is on the order of 100 μm, the width 10 μm, and the thickness 1 μm). The details of the model are shown in the Fig 1.

A Cartesian coordinate system *O*−*xyz* is established on the plate, as shown in Fig 1. The *Oxy* plane is the middle plane of the plate, and the displacement in the *x*, *y*, and *z* directions is represented as u, v, and w, respectively. we assume that the length of the plate in the x and y directions is *a* and *b* and the thickness is *H* (in the *z* direction). It is given a hypothesis that concentrated masses are attached to the upper and lower surfaces of the plate at ±*H* / 2 on the z axis and that the i^th^ particle has the coordinates (*x*_*i*_,*y*_*i*_,±*H* / 2). The Poisson's ratio of the plate is *μ*, the Young’s modulus is *E*, the density is *ρ*, and the mass of the i^th^ particle is *M*_*i*_.

### A. General Theory

In this section we present the theory of studying on dynamic behavior of plate-like micro-cantilever with multi-particles attached. This theoretical framework is applicable to thin plates of arbitrary plan view and uniform thickness exhibiting small deflections, where the effects of in-plane loading on the transverse (out-of-plane) deflections are negligible [13,14,15]. Since the sizes of the particles are negligible compared with the size of the plate, they can be regarded as concentrated masses. So only their kinetic energy and not their potential energy is considered in the analysis [8].

The Poisson-Kirchhoff theorem of thin plates is applied to establish the strain-displacement relationship (geometrical equation) and the stress-strain relationship (physical equation) of the micro-cantilever plate.

For small deformation and displacement, higher-order terms are negligible, and the following six equations are derived based on geometric principles [16]:

$$\begin{array}{lll} \varepsilon_{x}=\frac{\partial u}{\partial x}, & \varepsilon_{y}=\frac{\partial v}{\partial y}, & \varepsilon_{z}=\frac{\partial w}{\partial z}\\ \gamma_{yz}=\frac{\partial v}{\partial z}+\frac{\partial w}{\partial y}, & \gamma_{zx}=\frac{\partial w}{\partial x}+\frac{\partial u}{\partial z}, & \gamma_{xy}=\frac{\partial u}{\partial y}+\frac{\partial v}{\partial x} \end{array}$$ (1)

where *ε* represents unit elongation, and *γ* represents shear strain.

As the plate under study belongs to the Poisson-Kirchhoff theorem of thin plates, according to straight line assumption, we have *γ*_*yz*_ = *γ*_*zx*_ = 0. Introducing the expressions of *γ*_*yz*_ and *γ*_*zx*_ in Eq (1) yields.

$$\begin{array}{l} u(x,y,z;t)=u_{0}(x,y,0;t)-z\frac{\partial w}{\partial x}\\ v(x,y,z;t)=v_{0}(x,y,0;t)-z\frac{\partial w}{\partial y} \end{array}$$ (2)

where *u*_0_ and *v*_0_ are the displacements of the middle plane. According to the theory [11], no deformations occur in the middle plane, so both *u*_0_ and *v*_0_ are zero. Then Eq (2) is inserted into Eq (1) and the above analysis yields the strain-displacement relationship of the Poisson-Kirchhoff thin plate (geometric equation):

$$\varepsilon_{x}=-z\frac{\partial^{2}w}{\partial x^{2}},\;\varepsilon_{y}=-z\frac{\partial^{2}w}{\partial y^{2}},\;\gamma_{xy}=-2z\frac{\partial^{2}w}{\partial x\partial y}$$ (3)

The general form of the physical equation of an isotropic elastic material is[16]

$$\begin{array}{l} \;\varepsilon_{x}=\frac{1}{E}\left[\sigma_{x}-\mu \left(\sigma_{y}+\sigma_{z}\right)\right]\\ \;\varepsilon_{y}=\frac{1}{E}\left[\sigma_{y}-\mu \left(\sigma_{z}+\sigma_{x}\right)\right]\\ \;\varepsilon_{z}=\frac{1}{E}\left[\sigma_{z}-\mu \left(\sigma_{x}+\sigma_{y}\right)\right]\\ \gamma_{xy}=\frac{\tau_{xy}}{G},\;\gamma_{yz}=\frac{\tau_{yz}}{G},\;\gamma_{zx}=\frac{\tau_{zx}}{G} \end{array}$$ (4)

According to the above analysis, we have *γ*_*yz*_ = *γ*_*zx*_ = 0. We can assume the normal stress perpendicular to the middle plane is neglected [16], in other words, the normal stress perpendicular to the middle plane is zero (*σ*_*z*_ = 0). Inserting these values into Eq (4), and combining with G = E / (2(1 + *μ*)), yields

$$\begin{array}{lll} \sigma_{x} & = & \frac{E}{1-\mu^{2}}\left(\varepsilon_{x}+\mu \varepsilon_{y}\right)\\ \sigma_{y} & = & \frac{E}{1-\mu^{2}}\left(\varepsilon_{y}+\mu \varepsilon_{x}\right)\\ \tau_{xy} & = & G\gamma_{xy} \end{array}$$ (5)

### B. Energy of the micro-cantilever plate with concentrated masses attached

First, the energy of the plate is calculated. The potential energy of the deformation of an isotropic elastic body is

$$U_{C}=\underset{V}{∭}\frac{1}{2}\left(\sigma_{x}\varepsilon_{x}+\sigma_{y}\varepsilon_{y}+\sigma_{z}\varepsilon_{z}+\tau_{xy}\gamma_{xy}+\tau_{yz}\gamma_{yz}+\tau_{zx}\gamma_{zx}\right)\text{d}V$$ (6)

For the Poisson-Kirchhoff thin plate in this study, we have *σ*_*z*_ = *τ*_*yz*_ = *τ*_*zx*_ = 0, so Eq (6) can be further simplified. Substituting Eqs (3) and (5) into Eq (6) yields

$$\begin{array}{l} U_{C}=\int_{0}^{a}\int_{0}^{b}\int_{\;-\frac{H}{2}}^{\;\frac{H}{2}}\frac{1}{2}\left(\sigma_{x}\varepsilon_{x}+\sigma_{y}\varepsilon_{y}+\tau_{xy}\gamma_{xy}\right)\text{d}x\text{d}y\text{d}z\\ \;=\int_{0}^{a}\int_{0}^{b}\int_{\;-\frac{H}{2}}^{\;\frac{H}{2}}\frac{1}{2}\left(\frac{E}{1-\mu^{2}}\left(\varepsilon_{x}+\mu \varepsilon_{y}\right)\varepsilon_{x}+\frac{E}{1-\mu^{2}}\left(\varepsilon_{y}+\mu \varepsilon_{x}\right)\varepsilon_{y}+G\gamma_{xy}\gamma_{xy}\right)\text{d}x\text{d}y\text{d}z\\ \;=\int_{0}^{a}\int_{0}^{b}\int_{\;-\frac{H}{2}}^{\;\frac{H}{2}}\frac{1}{2}\frac{Ez^{2}}{1-\mu^{2}}[\left(\frac{\partial^{2}w}{\partial x^{2}}+\mu \frac{\partial^{2}w}{\partial y^{2}}\right)\frac{\partial^{2}w}{\partial x^{2}}+\left(\frac{\partial^{2}w}{\partial y^{2}}+\mu \frac{\partial^{2}w}{\partial x^{2}}\right)\times \\ \;\frac{\partial^{2}w}{\partial y^{2}}+\frac{\left(1-\mu \right)}{2}{\left(-2\frac{\partial^{2}w}{\partial x\partial y}\right)}^{2}]\text{d}x\text{d}y\text{d}z\\ \;=\frac{D}{2}\int_{0}^{a}\int_{0}^{b}[{\left(\frac{\partial^{2}w}{\partial x^{2}}\right)}^{2}+{\left(\frac{\partial^{2}w}{\partial y^{2}}\right)}^{2}+2\mu \frac{\partial^{2}w}{\partial x^{2}}\frac{\partial^{2}w}{\partial y^{2}}+2(1-\mu ){\left(\frac{\partial^{2}w}{\partial x\partial y}\right)}^{2}]\text{d}x\text{d}y \end{array}$$ (7)

where *D* is the bending stiffness of the plate and can be expressed as[17]

$$D=\frac{EH^{3}}{12\left(1-\mu^{2}\right)}$$ (8)

Eq (7) is the potential energy due to the deformation of the plate derived with classical mechanics. When the effect of the size of the plate is considered, the bending stiffness of the plate is changed and is represented by *D*′[17]

$$D\prime =\frac{EH^{3}}{12\left(1-\mu^{2}\right)}+\frac{EHl^{2}}{2\left(1+\mu \right)}$$ (9)

Accordingly, the potential energy due to the deformation of the plate is

$$U_{C}=\frac{D\prime }{2}\int_{0}^{a}\int_{0}^{b}[{\left(\frac{\partial^{2}w}{\partial x^{2}}\right)}^{2}+{\left(\frac{\partial^{2}w}{\partial y^{2}}\right)}^{2}+2\mu \frac{\partial^{2}w}{\partial x^{2}}\frac{\partial^{2}w}{\partial y^{2}}+2(1-\mu ){\left(\frac{\partial^{2}w}{\partial x\partial y}\right)}^{2}]\text{d}x\text{d}y$$ (10)

Comparing Eqs (7) and (10) shows that their only difference lies in the bending stiffness.

If only the transverse bending vibration of the plate (movement in the *z* direction, that is *w*) is considered and the movements in the plane of the plate (movements in the *x* and *y* directions, that are *u* and *v*) are neglected, the kinetic energy of the plate is

$$\begin{array}{l} T_{C}=\underset{V}{∭}\frac{1}{2}\text{dk}\cdot v_{z}{}^{2}\text{ = }\int_{0}^{a}\int_{0}^{b}\int_{\;-\frac{H}{2}}^{\;\frac{H}{2}}\frac{1}{2}{\left(\frac{\partial w}{\partial t}\right)}^{2}\cdot \rho \text{d}x\text{d}y\text{d}z\\ \;=\frac{1}{2}\rho H\int_{0}^{a}\int_{0}^{b}{\left(\frac{\partial w}{\partial t}\right)}^{2}\text{d}x\text{d}y \end{array}$$ (11)

After the energy of the plate is obtained, the energy of the attached concentrated masses must be derived. As stated above, it is assumed that *N* numbers of particles that can be taken as concentrated masses are attached to the upper (or lower) surface of the micro-cantilever plate. As the particles can be taken as concentrated masses, their sizes are negligible compared with the size of the plate, so the particles have no strain energy resulting from deformation, and only the kinetic energy of the particles needs to be considered.

The *i*^th^ particle has mass *M*_*i*_ and coordinates (*x*_*i*_,*y*_*i*_,*z*_*i*_), where *z*_*i*_ = *H*/ 2 (when the particle is attached to the upper surface of the plate) or *z*_*i*_ = −*H*/ 2 (when the particle is attached to the lower surface of the plate). Its velocity is $v_{z}^{i}=\frac{\partial w\left(x_{i},y_{i},z_{i}\right)}{\partial t}$ (only the movement of the particle in the direction of the transverse bending vibration of the plate is considered (or in the *z* direction,*w*)), so the total kinetic energy of *N* numbers of particles is

$$T_{P}=\sum_{i=1}^{N}\frac{1}{2}M_{i}{\left(v_{z}^{i}\right)}^{2}=\sum_{i=1}^{N}\frac{1}{2}M_{i}{\left[\frac{\partial w\left(x_{i},y_{i},z_{i}\right)}{\partial t}\right]}^{2}$$ (12)

Now, the expression of the total energy of the micro-cantilever plate with *N* attached particles is as follows:

$$U=U_{C},\;T=T_{C}+T_{P}$$ (13)

### C. Dynamic Equations

First, the vibration profiles of the plate are approximated. In this study, combinations of the beam functions satisfying the boundary conditions are used to approximate the vibration profiles of the plate, and the displacement of the vibration in the *z* direction is expressed as

$$W\left(x,y,t\right)=\sum_{\text{m}=1}^{\infty }\sum_{n=1}^{\infty }c_{mn}W_{m}^{x}(x)\cdot W_{n}^{y}\left(y\right)\cdot \text{sin}\omega_{mn}t$$ (14)

Eq (14) does not contain *z*, as it is assumed that no deformation occurs in the *z* direction, i.e., each point on the normal of the middle plane has uniform displacement *w* in the *z* direction. In this equation, *m* and *n* represent the order of beam functions included in the vibration profile along *x* and *y* directions, respectively. *c*_*mn*_ is an unknown weighting coefficient. sin *ωt* is the harmonic function. $W_{\text{m}}^{x}(x)$ is the m^th^ vibration-profile beam function corresponding to the boundary conditions along *x* direction, and $W_{n}^{y}\left(y\right)$ is the n^th^ vibration-profile beam function corresponding to the boundary conditions along *y* direction. The detailed expressions are shown in Table 1.

**Table 1:** Beam vibration-profile functions expression in the different boundary conditions.

left boundary condition *x* = 0	right boundary condition *x* = *a*	$W_{m}^{x}(x)$
S	S	$\text{sin}\frac{m\pi }{a}x$
C	C	(cosh*α*_*m*_*x* − cos*α*_*m*_*x*) − *C*_*m*_(sinh*α*_*m*_*x* − sin*α*_*m*_*x*)
F	F	$\begin{array}{l} \;W_{1}^{x}=1,\;W_{2}^{x}=\sqrt{3}\left(1-\frac{2x}{a}\right)\\ W_{m}^{x}=\left(\text{cosh}\alpha_{m}x+\text{cos}\alpha_{m}x\right)-C_{m}\left(\text{sinh}\alpha_{m}x+\text{sin}\alpha_{m}x\right)\;\left(m\geq 3\right) \end{array}$
C	S	(cosh*α*_*m*_*x* − cos*α*_*m*_*x*) − *C*_*m*_(sinh*α*_*m*_*x* − sin*α*_*m*_*x*)
C	F	(cosh*α*_*m*_*x* − cos*α*_*m*_*x*) − *C*_*m*_(sinh*α*_*m*_*x* − sin*α*_*m*_*x*)
F	S	$\begin{array}{l} \;W_{1}^{x}=\sqrt{3}\left(1-\frac{x}{a}\right)\\ W_{m}^{x}=\left(\text{cosh}\alpha_{m}x+\text{cos}\alpha_{m}x\right)-C_{m}\left(\text{sinh}\alpha_{m}x+\text{sin}\alpha_{m}x\right)\;\left(m\geq 2\right) \end{array}$

The expression describing the coefficient *C*_*m*_ in Table 1 is shown in Table 2, and the expression describing the coefficient in Tables 1 and 2 is *α*_*m*_ = *α*_*m*_*a* / *a*, where *α*_*m*_*a* is calculated in Table 3. Note that the vibration-profile beam function in the *y* direction, $W_{n}^{y}(y)$, has the same form as the vibration-profile beam function in the *x* direction, $W_{m}^{x}(x)$, so only $W_{m}^{x}(x)$ is listed in Tables 1–3. The cantilever plate in this study has the following boundary conditions: free-free in the x direction (F-F); in the y direction, fixed at *y* = 0(C), and free (F) at *y* = *b*.

**Table 2:** The expression for the coefficient C_m_ of beam vibration-profile functions is shown.

left boundary condition *x* = 0	right boundary condition *x* = *a*	*C*_*m*_	left boundary condition *x* = 0	right boundary condition *x* = *a*	*C*_*m*_
S	S	——	C	S	$\frac{\text{cosh}\alpha_{m}a+\text{cos}\alpha_{m}a}{\text{sinh}\alpha_{m}a+\text{sin}\alpha_{m}a}$
C	C	$\frac{\text{cosh}\alpha_{m}a-\text{cos}\alpha_{m}a}{\text{sinh}\alpha_{m}a-\text{sin}\alpha_{m}a}$	C	F	$\frac{\text{cosh}\alpha_{m}a+\text{cos}\alpha_{m}a}{\text{sinh}\alpha_{m}a+\text{sin}\alpha_{m}a}$
F	F	$\frac{\text{cosh}\alpha_{m}a-\text{cos}\alpha_{m}a}{\text{sinh}\alpha_{m}a-\text{sin}\alpha_{m}a}\left(m\geq 3\right)$	F	S	$\frac{\text{cosh}\alpha_{m}a\text{+cos}\alpha_{m}a}{\text{sinh}\alpha_{m}a\text{+sin}\alpha_{m}a}\left(m\geq 2\right)$

**Table 3:** The coefficient *α*_*m*_*a* is given in the different boundary conditions.

Boundary conditions		*α*_*m*_*a*					
*x* = 0	*x* = *a*	*m* = 1	*m* = 2	*m* = 3	*m* = 4	*m* = 5	*m*≥6
S	S	*π*	2*π*	3*π*	4*π*	5*π*	*mπ*
C	C	4.73004	7.85320	10.9956	14.1372	17.2786	$\left(2m+1\right)\frac{\pi }{2}$
F	F	0	0	4.73004	7.8532	10.9956	$\left(2m-3\right)\frac{\pi }{2}$
C	S	3.9266	7.06858	10.2102	13.3518	16.4934	$\left(4m+1\right)\frac{\pi }{4}$
C	F	1.87510	4.69409	7.85476	10.9955	14.1372	$\left(2m-1\right)\frac{\pi }{2}$
F	S	0	3.9266	7.06858	10.2102	13.3518	$\left(4m-3\right)\frac{\pi }{4}$

F-F: free-free boundary conditions [18]

$$\begin{array}{l} \begin{array}{lll} W_{m}^{x}(x) & = & \left\{ \begin{array}{ll} 1 & m=1\\ \sqrt{3}\left(\frac{2}{a}x-1\right) & m=2\\ C_{m1}\left(\text{cosh}\alpha_{m}x+\text{cos}\alpha_{m}x\right)-C_{m2}\left(\text{sinh}\alpha_{m}x+\text{sin}\alpha_{m}x\right) & m\geq 3 \end{array}\right.\\ C_{m1} & = & \frac{\text{cosh}\alpha_{m}a-\text{cos}\alpha_{m}a}{\text{sinh}\alpha_{m}a\text{sin}\alpha_{m}a},\;C_{m2}=\frac{\text{sinh}\alpha_{m}a+\text{sin}\alpha_{m}a}{\text{sinh}\alpha_{m}a\text{sin}\alpha_{m}a} \end{array}\\ \alpha_{3}a=4.730,\;\alpha_{4}a=7.853,\;\alpha_{5}a=10.995,\;\alpha_{m}a=\left(2m-3\right)\frac{\pi }{2}\left(m>5\right) \end{array}$$ (15)

C-F: fixed-free boundary conditions [18]

$$\begin{array}{l} \begin{array}{ll} W_{m}^{x}(x) & =C_{m1}\left(\text{cosh}\alpha_{m}x-\text{cos}\alpha_{m}x\right)-C_{m2}\left(\text{sinh}\alpha_{m}x-\text{sin}\alpha_{m}x\right)\\ C_{m1} & =\frac{\text{cosh}\alpha_{m}a+\text{cos}\alpha_{m}a}{\text{sinh}\alpha_{m}a\text{sin}\alpha_{m}a},\;C_{m2}=\frac{\text{sinh}\alpha_{m}a-\text{sin}\alpha_{m}a}{\text{sinh}\alpha_{m}a\text{sin}\alpha_{m}a} \end{array}\\ \alpha_{1}a=1.875,\;\alpha_{2}a=4.694,\;\alpha_{3}a=7.854,\;\alpha_{m}a=\left(2m-1\right)\frac{\pi }{2}\left(m>3\right) \end{array}$$ (16)

Let *c*_*mn*_ sin *ω*_*mn*_*t* be *q*_*mn*_(*t*), which is chosen as the generalized coordinate, and the beam functions in the *x* and *y* directions are truncated to the *m*_0_^th^ and *n*_0_^th^ modes, respectively. Transforming Eq (14) into the form of multiplication of vectors yields

$$W\left(x,y,t\right)=W^{\text{T}}(x,y)q(t)$$ (17)

where **W**(*x*,*y*) and **q**(*t*) have the following expressions:

$$\begin{array}{l} \begin{array}{lll} W(x,y) & = & [W_{1}^{x}\cdot W_{1}^{y},\cdots ,W_{1}^{x}\cdot W_{n_{0}}^{y},W_{2}^{x}\cdot W_{1}^{y},\cdots ,W_{2}^{x}\cdot W_{n_{0}}^{y},\\ & & {\cdots W_{m}^{x}\cdot W_{n}^{y},\cdots ,W_{m_{0}}^{x}\cdot W_{1}^{y},\cdots ,W_{m_{0}}^{x}\cdot W_{n_{0}}^{y}]}^{\text{T}} \end{array}\\ q(t)={\left[q_{11},\cdots ,q_{1n_{0}},q_{21},\cdots ,q_{2n_{0}},\cdots ,q_{mn},\cdots ,q_{m_{0}1},\cdots ,q_{m_{0}n_{0}}\right]}^{\text{T}} \end{array}$$ (18)

The vibration-profile function of the plate**W**(*x*,*y*) constructed in this section does not contain the *z* coordinate. The expression describing the kinetic energy of the attached concentrated masses also shows that the location of the masses in the z direction does not affect their kinetic energy. Therefore, only the projected locations of the particles are required in the computation, i.e., the (*x*_*i*_,*y*_*i*_) coordinates.

Insert the vibration-profile of the plate into the elastic potential energy and kinetic energy of the micro-cantilever plate with concentrated masses attached. The expression of the energy can be separated and represented as a superposition of finite terms. The dynamic equations can then be derived using the Lagrange equations.

It is noted that the expression describing the energy of the micro-cantilever plate with attached concentrated masses includes the power and multiplication of $\frac{\partial^{2}w}{\partial x^{2}}$, $\frac{\partial^{2}w}{\partial y^{2}}$, $\frac{\partial^{2}w}{\partial x\partial y}$ and $\frac{\partial w}{\partial t}$, so their expressions are derived as follows:

$$\frac{\partial^{2}w}{\partial x^{2}}=\frac{\partial^{2}W^{\text{T}}}{\partial x^{2}}q,\;\frac{\partial^{2}w}{\partial y^{2}}=\frac{\partial^{2}W^{\text{T}}}{\partial y^{2}}q,\;\frac{\partial^{2}w}{\partial x\partial y}=\frac{\partial^{2}W^{\text{T}}}{\partial x\partial y}q,\;\frac{\partial w}{\partial t}=W^{\text{T}}\dot{q}$$ (19)

$$\begin{array}{l} {\left(\frac{\partial^{2}w}{\partial x^{2}}\right)}^{2}=q^{\text{T}}\frac{\partial^{2}W^{}}{\partial x^{2}}\frac{\partial^{2}W^{\text{T}}}{\partial x^{2}}q,\;{\left(\frac{\partial^{2}w}{\partial y^{2}}\right)}^{2}=q^{\text{T}}\frac{\partial^{2}W^{}}{\partial y^{2}}\frac{\partial^{2}W^{\text{T}}}{\partial y^{2}}q\\ \frac{\partial^{2}w}{\partial x^{2}}\frac{\partial^{2}w}{\partial y^{2}}=q^{\text{T}}\frac{\partial^{2}W^{}}{\partial x^{2}}\frac{\partial^{2}W^{\text{T}}}{\partial y^{2}}q,\;{\left(\frac{\partial^{2}w}{\partial x\partial y}\right)}^{2}=q^{\text{T}}\frac{\partial^{2}W^{}}{\partial x\partial y}\frac{\partial^{2}W^{\text{T}}}{\partial x\partial y}q\\ \;{\left(\frac{\partial w}{\partial t}\right)}^{2}={\dot{q}}^{\text{T}}W^{}W^{\text{T}}\dot{q} \end{array}$$ (20)

The Galerkin discrete energy is derived as follows:

$$\begin{array}{l} U_{C}=\frac{D}{2}\int_{0}^{a}\int_{0}^{b}[q^{\text{T}}\frac{\partial^{2}W^{}}{\partial x^{2}}\frac{\partial^{2}W^{\text{T}}}{\partial x^{2}}q+q^{\text{T}}\frac{\partial^{2}W^{}}{\partial y^{2}}\frac{\partial^{2}W^{\text{T}}}{\partial y^{2}}q+\\ \;2\mu q^{\text{T}}\frac{\partial^{2}W^{}}{\partial x^{2}}\frac{\partial^{2}W^{\text{T}}}{\partial y^{2}}q+2(1-\mu )q^{\text{T}}\frac{\partial^{2}W^{}}{\partial x\partial y}\frac{\partial^{2}W^{\text{T}}}{\partial x\partial y}q]\text{d}x\text{d}y \end{array}$$ (21)

$$T_{C}=\frac{1}{2}\rho H\int_{0}^{a}\int_{0}^{b}{\dot{q}}^{\text{T}}{W\;}^{}W^{\text{T}}\dot{q}\text{d}x\text{d}y$$ (22)

$$T_{P}=\sum_{i=1}^{N}\frac{1}{2}m_{i}{\left(v_{z}^{i}\right)}^{2}=\sum_{i=1}^{N}\frac{1}{2}m_{i}{\dot{q}}^{\text{T}}W{\left(x_{i},y_{i},z_{i}\right)}^{}W^{\text{T}}\left(x_{i},y_{i},z_{i}\right)\dot{q}$$ (23)

$$\begin{array}{llllll} K_{1} & = & \frac{D}{2}\int_{0}^{a}\int_{0}^{b}\frac{\partial^{2}W^{}}{\partial x^{2}}\frac{\partial^{2}W^{\text{T}}}{\partial x^{2}}\text{d}x\text{d}y, & K_{2} & = & \frac{D}{2}\int_{0}^{a}\int_{0}^{b}\frac{\partial^{2}W^{}}{\partial y^{2}}\frac{\partial^{2}W^{\text{T}}}{\partial y^{2}}\text{d}x\text{d}y\\ K_{3} & = & D\mu \int_{0}^{a}\int_{0}^{b}\frac{\partial^{2}W^{}}{\partial x^{2}}\frac{\partial^{2}W^{\text{T}}}{\partial y^{2}}\text{d}x\text{d}y, & K_{4} & = & D(1-\mu )\int_{0}^{a}\int_{0}^{b}\frac{\partial^{2}W^{}}{\partial x\partial y}\frac{\partial^{2}W^{\text{T}}}{\partial x\partial y}\text{d}x\text{d}y\\ M_{0} & = & \frac{1}{2}\rho H\int_{0}^{a}\int_{0}^{b}{W\;}^{}W^{\text{T}}\text{d}x\text{d}y, & M_{i} & = & \frac{1}{2}m_{i}W{(x_{i},y_{i},z_{i})\;}^{}W^{\text{T}}\left(x_{i},y_{i},z_{i}\right) \end{array}$$ (24)

Eqs (22), (23) and (24) can be simplified as follows:

$$U_{C}=q^{\text{T}}K_{1}q+q^{\text{T}}K_{2}q+q^{\text{T}}K_{3}q+q^{\text{T}}K_{4}q$$ (25)

$$T_{C}={\dot{q}}^{\text{T}}M_{0}\dot{q}$$ (26)

$$T_{P}=\sum_{i=1}^{N}\frac{1}{2}m_{i}{\left(v_{z}^{i}\right)}^{2}={\dot{q}}^{\text{T}}\left(\sum_{i=1}^{N}M_{i}\right)\dot{q}$$ (27)

The vector form of the Lagrange equation of the micro-cantilever plate with attached concentrated masses is

$$\frac{\text{d}}{\text{d}t}\left[\frac{\partial \left(T_{C}+T_{P}\right)}{\partial \dot{q}}\right]-\frac{\partial }{\partial q}\left(T_{C}+T_{P}\right)+\frac{\partial U_{C}}{\partial q}=Q$$ (28)

where **q** is the generalized coordinate vector, and **Q** is the generalized force vector. As free vibration is studied in this paper, we set **Q** = **0** to obtain the natural frequencies and modes of the micro-cantilever plate with attached concentrated masses.

The detailed expressions of dynamic equation are derived as follows:

$$\begin{array}{l} \frac{\partial \left(T_{C}+T_{P}\right)}{\partial \dot{q}}=\left(M_{0}^{\text{T}}\text{+}M_{0}\right)\dot{q}+\left[\left(\sum_{i=1}^{N}M_{i}^{\text{T}}\right)+\left(\sum_{i=1}^{N}M_{i}\right)\right]\dot{q}\\ \frac{\text{d}}{\text{d}t}\left[\frac{\partial \left(T_{C}+T_{P}\right)}{\partial \dot{q}}\right]=\left(M_{0}^{\text{T}}\text{+}M_{0}\right)\ddot{q}+\left[\left(\sum_{i=1}^{N}M_{i}^{\text{T}}\right)+\left(\sum_{i=1}^{N}M_{i}\right)\right]\ddot{q}\\ \frac{\partial }{\partial q}\left(T_{C}+T_{P}\right)=0\\ \frac{\partial U_{C}}{\partial q}=\left(K_{1}^{\text{T}}+K_{1}\right)q\text{+}\left(K_{2}^{\text{T}}+K_{2}\right)q\text{+}\left(K_{3}^{\text{T}}+K_{3}\right)q\text{+}\left(K_{4}^{\text{T}}+K_{4}\right)q \end{array}$$ (29)

Inserting Eq (29) into Eq (28) and arranging the terms yields

$$\begin{array}{l} \left[M_{0}^{\text{T}}\text{+}M_{0}+\left(\sum_{i=1}^{N}M_{i}^{\text{T}}\right)+\left(\sum_{i=1}^{N}M_{i}\right)\right]\ddot{q}+\\ \left(K_{1}^{\text{T}}+K_{1}\text{+}K_{2}^{\text{T}}+K_{2}\text{+}K_{3}^{\text{T}}+K_{3}\text{+}K_{4}^{\text{T}}+K_{4}\right)q=0 \end{array}$$ (30)

Eq (30) shows that **K**_1_, **K**_2_, **K**_4_, **M**_0_, and **M**_*i*_ are symmetric matrices, so they have the following property: $M_{0}^{\text{T}}\text{+}M_{0}\text{ = }2M_{0}$(the same for the other matrices), and Eq (30) can be simplified as follows:

$$2\left[M_{0}+\left(\sum_{i=1}^{N}M_{i}\right)\right]\ddot{q}+\left(2K_{1}\text{+2}K_{2}\text{+}K_{3}^{\text{T}}+K_{3}\text{+}2K_{4}\right)q=0$$ (31)

Eq (31) is the dynamic equation of free vibration of the micro-cantilever plate with attached concentrated masses (without considering the effect of size). When the effect of size is considered, the dynamic equation is similar to Eq (31), only with Eq (9) as the bending stiffness.

The general solution of ordinary differential equations has the following form:

$$q=ce^{\lambda t}$$ (32)

Inserting the above equation into Eq (31) yields

$$2\left[M_{0}+\left(\sum_{i=1}^{N}M_{i}\right)\right]c\lambda^{2}e^{\lambda t}\ddot{q}+\left(2K_{1}\text{+2}K_{2}\text{+}K_{3}^{\text{T}}+K_{3}\text{+}2K_{4}\right)ce^{\lambda t}=0$$ (33)

*e*^*λt*^ can be removed because *e*^*λt*^ ≠ 0, i.e.,

$$\left[\lambda^{2}\cdot 2\left(M_{0}+\sum_{i=1}^{N}M_{i}\right)+\left(2K_{1}\text{+2}K_{2}\text{+}K_{3}^{\text{T}}+K_{3}\text{+}2K_{4}\right)\right]c=0$$ (34)

Because the coefficient vector **c** ≠ **0**, we have

$$\left|\lambda^{2}\cdot 2\left(M_{0}+\sum_{i=1}^{N}M_{i}\right)+\left(2K_{1}\text{+2}K_{2}\text{+}K_{3}^{\text{T}}+K_{3}\text{+}2K_{4}\right)\right|=0$$ (35)

Eqs (34) and (35) are the characteristic equation and frequency equation of the system, respectively.

## Results and Discussion

To verify the efficacy of the dynamic model proposed in this paper, two comparisons are conducted with the results of published literatures.

**Fig 2:**
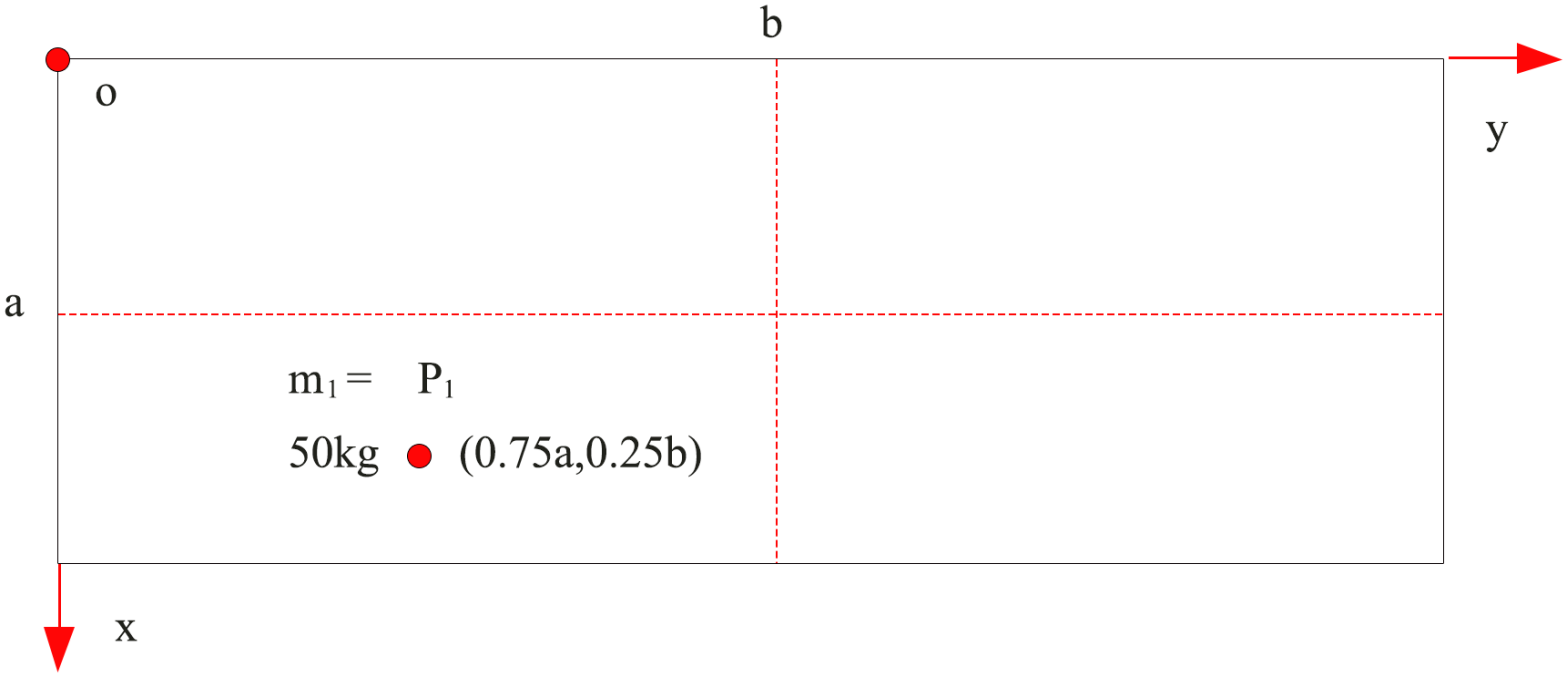
Diagrammatic sketch of the parameters of the attached mass, *P*_1_ (50kg, 0.75a, 0.25b).

The first comparison is made using the result computed with classical mechanics (the effect of size is not considered). We adopt the plate model that is shown by M1. The boundary condition is assumed as simply supported at four edges (SSSS). The geometrical parameter and material parameter of the plate are shown in Table 4. The mass is attached to the plate which is shown in Fig 2. The parametres of the attached masses are as follows: (*x*_*i*_,*y*_*i*_) = (0.75*a*,0.25*b*) and *m*_1_ = 50 kg. The bending stiffness in Eq (8) is adopted because we use classical mechanics. The frequency can be calculated by Eq (35), which is compared with the one in the Reference[19]. The comparison results are shown in Table 5. The comparisons show that the frequencies computed using the equation in this paper are agree very well with the computation results of reference [19], both within 0.5% error compared with the exact value. This agreement and accuracy indicate that the equation derived in this paper for calculating the frequencies of plates with attached masses is correct and has sufficient precision. The precision is enough in practical engineering application.

**Table 4:** Geometric and material parameters of the plates studied in this paper.

Dimension parameter	M1	M2	M3	Material parameter	M1	M2	M3
Length *a*	2 m	50 *μ* m	29 *μ* m	*E* (GPa)	205.1	50	160
Width *b*	2 m	50 *μ* m	87 *μ* m	Poisson's ratio	0.3	0.33	0.27
Thickness *H*	0.005 m	5 *μ* m	2 *μ* m	Density (kg/m^3^)	7850	2700	2320

**Table 5:** Natural frequencies of the M1 plate simply supported at four edges (comparison with classical mechanics).

Method	Natural frequencies (rad/s)				
	*ω*_1_	*ω*_2_	*ω*_3_	*ω*_4_	*ω*_5_
FEM^[^^19^^]^	32.503	63.913	97.13	130.077	182.947
ANCM^[^^19^^]^	31.814	63.232	95.415	127.616	180.593
Exact^[^^19^^]^	31.825	63.318	95.415	127.741	180.677
Present	31.854	63.550	95.415	128.073	180.891
Error (%)	0.090	0.367	0.000	0.260	0.119

The second comparison is made using the result computed with the DQ method and the Rayleigh-Ritz method (the effect of size is considered). We adopt the plate model that is shown by M2. The boundary condition is assumed as simply supported at four edges (SSSS). The geometrical parameter and material parameter of the plate are shown in Table 4. The character length of *l* is 3 *μm*. The bending stiffness in Eq (9) is adopted while the frequency is calculated in Eq (35). We made a comparison between the computed result and the result of Reference [17]. The comparison result is shown in Table 6. The results obtained from the method proposed in this paper agree very well with the those computed by using the DQ method and the Rayleigh-Ritz method [17]. This agreement reveals that the equation reported in this paper may be used to analyze the free vibration of micro-plates for which the size of the effect needs to be considered.

**Table 6:** Natural frequencies of the M2 plate simply supported at four edges (comparison with the DQ method and the Rayleigh-Ritz method; based on corrected Cosserat’s theorem (the effect of size is considered)).

Mode	Reference[17](*f* / 10^7^ Hz)		(*f* / 10^7^ Hz)	
	DQ method	Rayleigh-Ritz method	Frequency	Modal combination(m, n)
1	1.289	1.292	1.293	(1, 1)
2	5.154	5.171	5.174	(2, 2)
3	11.59	11.63	11.64	(3, 3)

**Fig 3:**
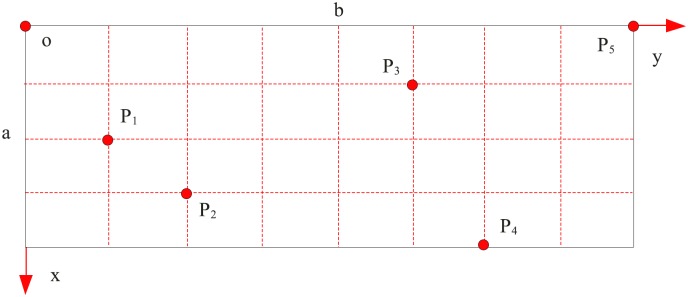
Locations of the attached masses. Five points are choosing on the plate, there mass and location are different.

Finally, we adopt the equation derived in this paper for calculating the natural frequencies of micro-cantilever plates with attached masses. we choose a type of rectangular micro-cantilever plate, which is shown as the plate M3 in Table 4. The cantilever length, width, thickness, E, Poisson's ratio and density are approximately 29*μ*m, 87*μ*m, 2*μ*m, 160GPa, 0.27 and 2320kg/m^3^, setting *l* = 0.365 *μ*m imposes the characteristic length. Fig 3 shows the locations of the masses, and the parameters of the masses are listed in Table 7. Then, the natural frequencies of the plate with 1–5 attached masses are computed, and the results are shown in Table 8.

**Table 7:** Location and mass of the attached mass on the plate.

Attached mass	*P*_1_	*P*_2_	*P*_3_	*P*_4_	*P*_5_
Location	(0.5*a*, 0.125*b*)	(0.75*a*, 0.25*b*)	(0.25*a*, 0.625*b*)	(*a*, 0.75*b*)	(0, *b*)
Mass	*m*_1_ = 0.05*m*_0_	*m*_1_ = 0.1*m*_0_	*m*_1_ = 0.15*m*_0_	*m*_1_ = 0.2*m*_0_	*m*_1_ = 0.25*m*_0_

**Table 8:** Natural frequencies of the plate-like micro-cantilever beam with attached masses from *P*_1_ *to P*_5_ for the first five modes.

Attached mass	*f*_1_	*f*_2_	*f*_3_	*f*_4_	*f*_5_
*P*_1_	3.8823E+05	2.4210E+06	2.4223E+06	6.7387E+06	7.6039E+06
*P*_1_, *P*_2_	3.8751E+05	2.3231E+06	2.4221E+06	6.0400E+06	7.4270E+06
*P*_1_, *P*_2_, *P*_3_,	3.6201E+05	2.1220E+06	2.3386E+06	5.6949E+06	7.4267E+06
*P*_1_, *P*_2_, *P*_3_, *P*_4_	3.1706E+05	1.6566E+06	2.1992E+06	5.2193E+06	7.4145E+06
*P*_1_, *P*_2_, *P*_3_, *P*_4_, *P*_5_	2.4472E+05	1.1501E+06	1.9518E+06	4.5446E+06	6.6338E+06

In the following, some studies about parameters are carried out by using the dynamic model which is attached in the masses on the plate M3 in Table 4. The locations and the parameters are showed in Fig 3 and in Table 7.

**Fig 4:**
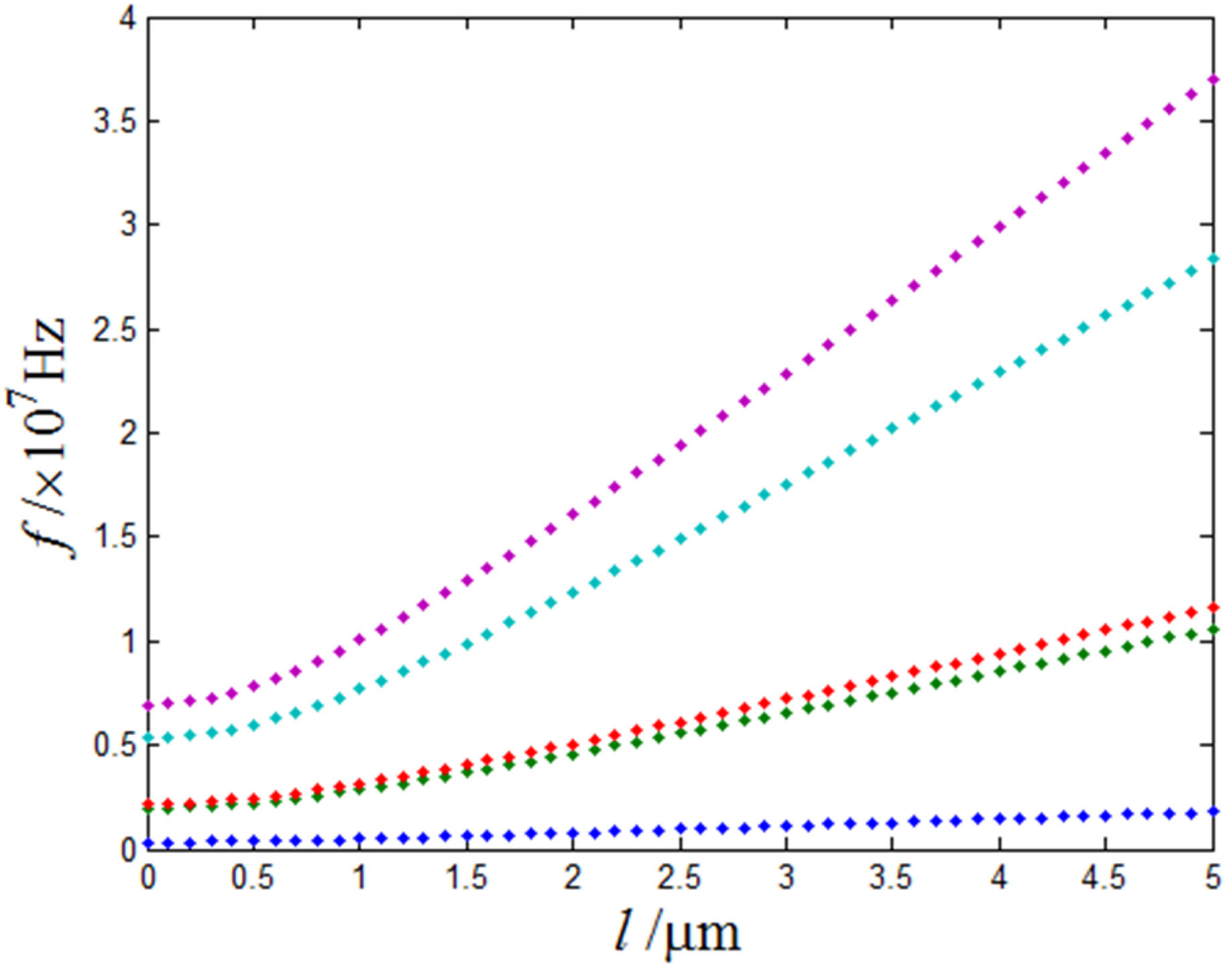
Different characteristic length for the first-five modes as a change of the micro-cantilever plate’s resonant frequency, l = 0μm-5μm.

Fig 4 shows that the variation of first-five resonant frequencies of the micro-cantilever plate attached five points with characteristic length. From this figure, it is seen that the change of the characteristic length causes the change of the micro-cantilever plate’s resonance frequency. When the vibration mode is different, the degree of frequency variation is also different. When the vibration modal is constant and characteristic length is small, the change of the micro-cantilever plate's resonance frequency is not obvious. When the characteristic length is big, the effect of size is obvious. With the increase of the characteristic length, the resonance frequency has increased dramatically.

**Fig 5:**
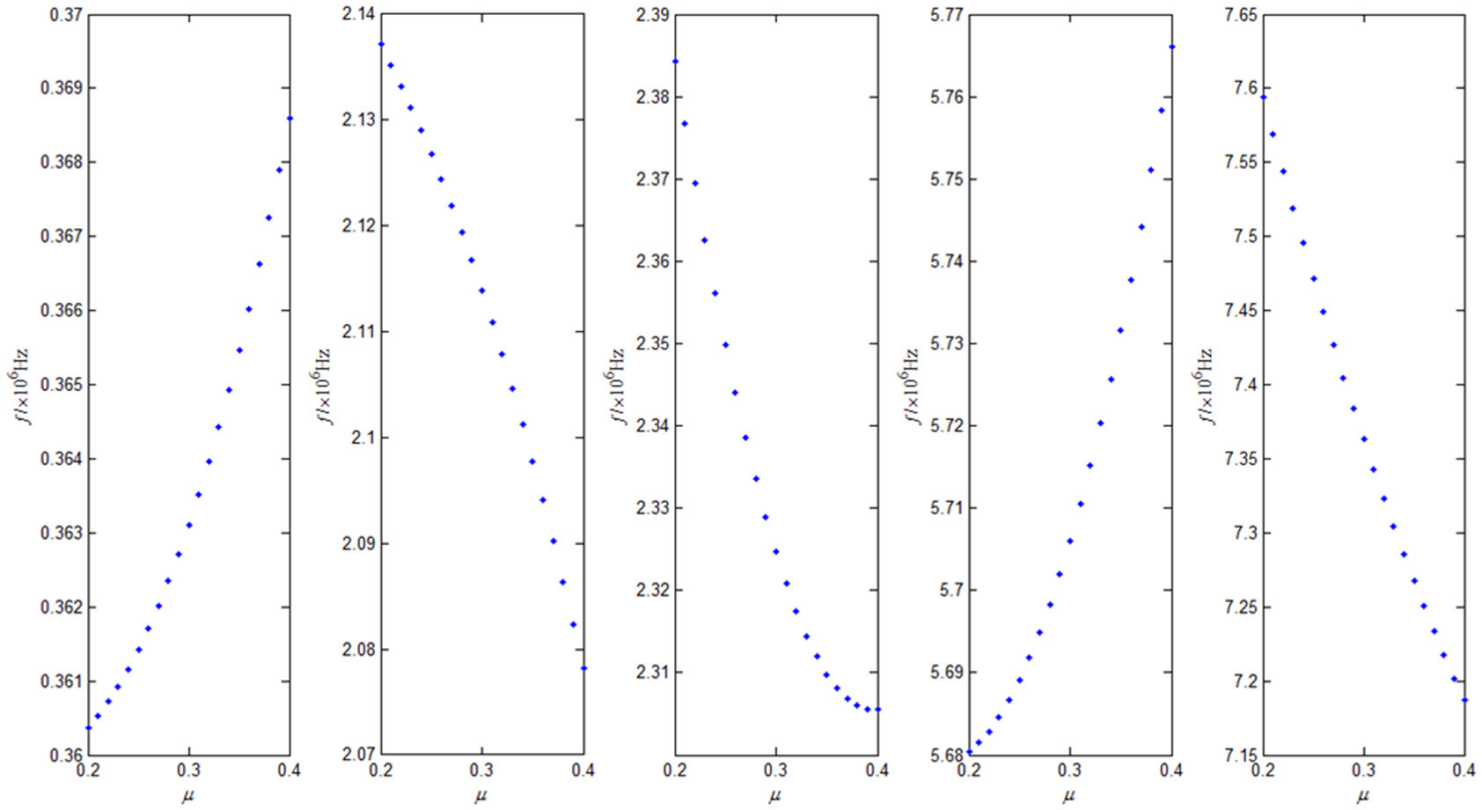
Different Poisson’s ratio for the first five modes as a change of the micro-cantilever plate’s resonant frequency, μ = 2–4.

We analyzed different Poisson's ratio and the quality of the five points distribution for the first five modes as a change of the micro-cantilever plate’s resonant frequency. From the Fig 5, it can be observed that different Poisson's ratio and points distribution will cause the change of the micro-cantilever plate’s resonance frequency, and each order resonance frequency’s change is different. Some resonance frequency reduce with the increase of the poisson's ratio, others are higher, but the change of the higher resonance modes are significant than the lower resonance modes.

**Fig 6:**
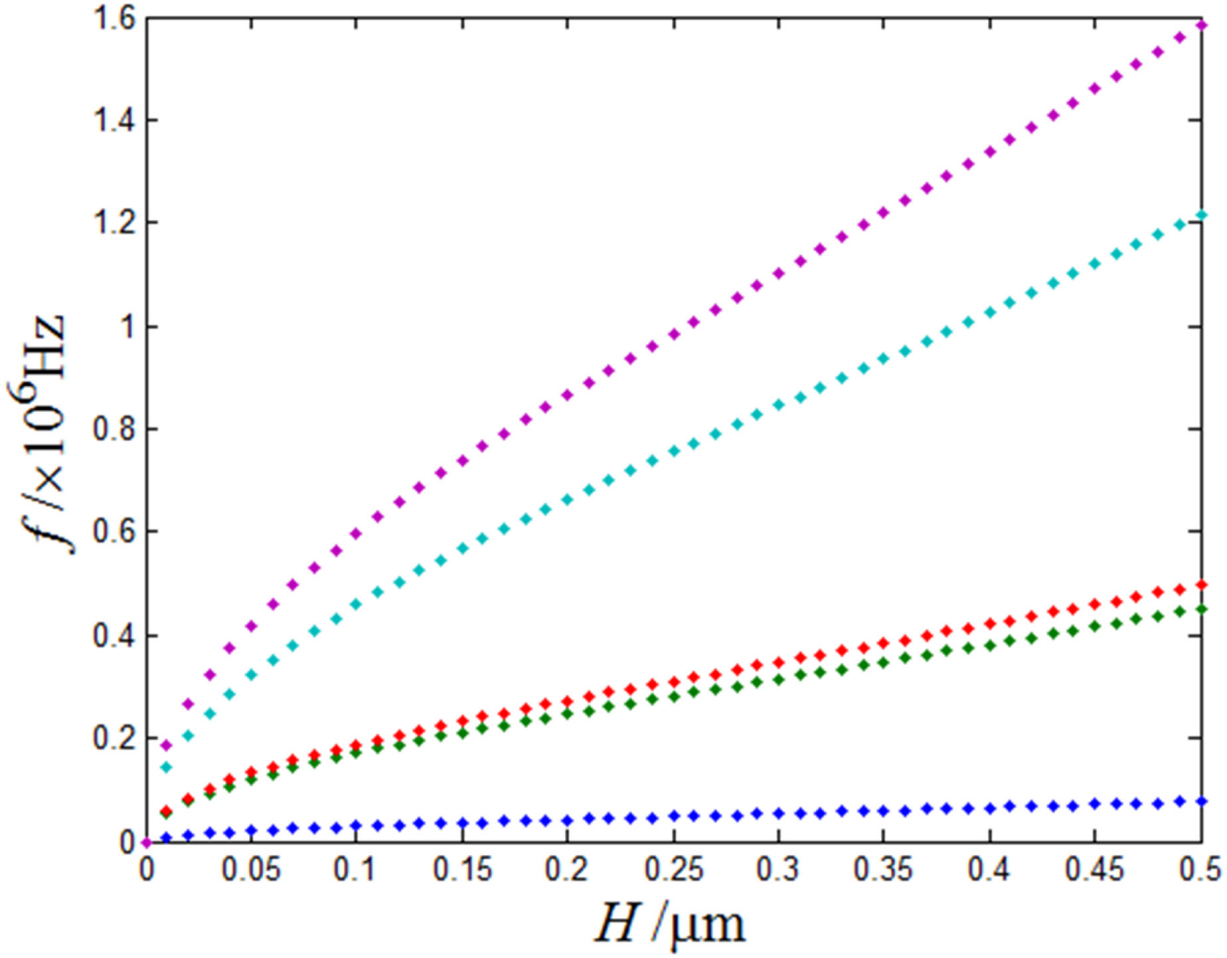
Different thickness of the plate for the first five modes as a change of the micro-cantilever plate’s resonant frequency, H = 0μm-5μm.

Fig 6 shows that the change of first-five resonant frequencies with different thicknesses of the plate and the qualities of five points distribution. From this figure, it is observed that the increase of the thickness will cause the increase of the micro-cantilever plate’s resonance frequency, and the increase of the higher resonance frequency are significant than the lower resonance frequency. About plate thickness influence for resonant frequency, which can be explained from bending rigidity. When the thickness increase, the bending stiffness will increase. It cause the increase of the micro-cantilever plate’s resonance frequency.

## Conclusions

This paper studies the dynamic characteristics of a micro-cantilever plate with multiple concentrated masses attached. Classical mechanics (the effect of size is not considered) and the corrected Cosserat’s theorem (the effect of size is considered) are alternatively used to establish the kinetic energy and elastic potential energy of the micro-cantilever plate, including the energy of the particles. The Galerkin discretization and the Lagrang equations are used to establish the dynamic model of the micro-cantilever plate with multiple concentrated masses attached.

To verify the accuracy of the model, first, the proposed method is compared with the finite element method (FEM) and analytical and numerical combined method (ANCM). The frequencies of a plate whose boundary condition is SSSS with attached masses are computed by the three methods, and the results are in high accordance with one another. All errors are within 0.5% of the exact solution. This agreement suggests that the equation derived in this paper for calculating the frequencies of a plate with attached masses is accurate and has sufficient precision.

Next, the proposed method is compared with the DQ method and the Rayleigh-Ritz method. The frequencies of a plate simply supported at four edges are calculated by the three methods, and the results agree well with one another. This comparison shows that the method proposed in this paper can be applied to analyzing the free vibration of micro-plates when the effect of size is considered.

Finally, the equation derived in this paper for calculating the natural frequencies of micro-cantilever plates with attached masses is used to calculate the natural bending frequencies of a plate-like micro-cantilever beam with concentrated masses attached, and the results with 1–5 masses are presented. Also we analyzed different characteristic length, Poisson's ratio and thickness of the plate for the first five modes as a change of the micro-cantilever plate’s resonant frequency. According the analysis results, we can further optimize relevant parameter and obtain the more accurate results.
